# Evaluation of deep vein thrombosis prophylaxis use in a Northwest Ethiopian medical ward: an observational follow-up study

**DOI:** 10.3389/fmed.2025.1468190

**Published:** 2025-03-21

**Authors:** Samuel Berihun Dagnew, Tilaye Arega Moges, Fisseha Nigussie Dagnew, Abraham Nigussie Assefa, Sisay Sitotaw Anberbr, Adane Tsegaw Geremew, Getu Tesfaw Addis

**Affiliations:** ^1^Department of Clinical Pharmacy, College Health Sciences, Debre Tabor University, Debre Tabor, Ethiopia; ^2^Department of Social and Administrative Pharmacy, College of Health Sciences, Debre Tabor University, Debre Tabor, Ethiopia

**Keywords:** deep vein thrombosis, appropriateness of thromboprophylaxis, medical ward, Comprehensive Specialized Hospitals, Northwest Ethiopia

## Abstract

**Background:**

During hospitalization, a significant number of patients at risk of thromboembolism do not receive prophylaxis, despite established standards and viable procedures for preventing deep vein thrombosis (DVT). This study aimed to assess the appropriateness of vein thrombosis prophylaxis use among patients admitted to the medical ward of Debre Tabor Comprehensive Specialized Hospital (DTCSH) in Northwest Ethiopia.

**Methods:**

An observational follow-up study was conducted in the medical wards of Debre Tabor Comprehensive Specialized Hospital in Northwest Ethiopia to determine whether thromboprophylaxis was appropriately used, based on the Padua risk assessment tool. To identify factors associated with the occurrence of inappropriate thromboprophylaxis use, a binary logistic regression model was used. Statistical significance was considered when the *p*-value was <0.05, with a 95% confidence interval.

**Results:**

Among the 365 patients in the study, 21.37% received inappropriate thromboprophylaxis, while 78.63% received it correctly. Patients admitted to the ICU [AOR = 4.276, 95% CI: 1.878–16.134; *p* = 0.000], those who stayed for more than 6 days [AOR =6.192, 95% CI: 2.085–14.391; *p* = 0.000], and general practitioners [AOR = 1.816, 95% CI: 1.007–3.207; *p* = 0.048] were more likely to receive inappropriate thrombophylaxis.

**Conclusion:**

The appropriateness of DVT prophylaxis use was suboptimal, especially among the patients treated by general practitioners, those hospitalized in the intensive care unit, and those who stayed for more than a few days in the ward. Using an integrated risk stratification checklist is an effective way to promote the more rational use of DVT prophylaxis.

## Introduction

Deep vein thrombosis (DVT) continues to be a serious condition with a high mortality and morbidity rate (1). Hospitalized patients who are critically ill and have limited movement are at an increased risk of venous thromboembolism (VTE), which can lead to deep vein thrombosis (DVT) and pulmonary embolism (PE) (2). Between 50,000 and 200,000 of the 600,000 hospital admissions associated with DVT result in a pulmonary embolism (3). An estimated 10 million cases of hospital-related venous thromboembolism occur annually, making it a major source of illness and mortality worldwide (4). The occurrence of these complications can be reduced by healthcare personnel implementing effective prevention strategies, such as early mobilization and pharmaceutical prophylaxis. Furthermore, educating patients about symptom recognition and mobility maintenance can improve outcomes and reduce the impact of VTE (5).

In the absence of prevention, a significant number of medical patients experience an increased DVT rate (6). The occurrence of VTE often complicates the treatment plan for hospitalized patients (7). The risk of DVT and its associated consequences in hospitalized patients is considerably decreased when appropriate thromboprophylaxis, such as anticoagulant drugs or mechanical devices, is used (8, 9). However, in hospital settings, thromboprophylaxis is often either underappreciated or overused (10, 11). Negative effects, such as increased morbidity, mortality, and medical expenses, can result from the improper use of DVT prophylaxis—whether through underutilization or overutilization (12).

Inappropriate or non-existent DVT prevention in hospitalized patients can have fatal consequences. Patients with DVT are at a high risk of developing serious complications, including pulmonary embolism, which can lead to significant long-term health issues or even death (13). Furthermore, the development of DVT can lead to long-term complications, such as bleeding, recurrent venous thrombosis, chronic post-thrombotic syndrome (PTS) sequelae, persistent dyspnea following PE, and chronic thromboembolic pulmonary hypertension (14).

In the context of Ethiopia, only a small number of studies have evaluated whether DVT prophylaxis should be used among hospitalized patients in Ethiopia. Research from Jimma University Specialized Hospital revealed that only 37.3% of eligible patients received appropriate thromboprophylaxis (15). Another study conducted at Tikur Anbessa Specialized Hospital at Addis Ababa University revealed that 54.7% of patients received the recommended DVT prophylaxis (16).

The aim of this study was to assess the appropriateness of DVT prophylaxis use among patients admitted to the medical ward of Debre Tabor Comprehensive Specialized Hospital in Northwest Ethiopia. Understanding the fundamental causes of inadequate DVT prophylaxis is essential for formulating interventions that enhance patient outcomes. A detailed assessment of current practices and the identification of opportunities for improvement can help shape the development of targeted interventions to enhance patient safety and quality of care.

## Materials and methods

### Study setting, period, and design

The study was conducted at Debre Tabor Comprehensive Specialized Hospital (DTCSH), located in the South Gondar zone of the Amhara Region in Northwest Ethiopia, 100 km from Bahirdar and 666 from Addis Ababa. In the catchment area, it serves approximately 3 million people. The hospital has several departments, including internal medicine, pediatrics, obstetrics and gynecology, surgery, dentistry, psychiatry, ophthalmology, hospital pharmacy, dermatology, laboratory services, and an antiretroviral therapy clinic. In the medical wards of the hospital, an observational follow-up study design was used to evaluate the appropriateness of pharmacological prophylaxis against deep vein thrombosis between 18 March and 30 May 2024.

### Study participants and sampling technique

The sample size was calculated using the single population formula, $n=\;\frac{{\left(Z\alpha /2\right)}^{2}P\left(1-P\right)}{d^{2}}$ . The prevalence of inappropriate DVT prevention was obtained from a study conducted at UoCSH (17). A total sample size of 332 was calculated with a *p*-value of 31.6% (0.5), a marginal error (d) of 5%, a threshold of significance (*α*) of 0.05, Zα/2 = 1.96, and *q* = 1−*p*, based on prior research of this type. The final sample size was 365 individuals after accounting for a 10% contingency. All patients admitted to the DTCSH medical ward during the study period who met the inclusion criteria were included in the study. Patients who were readmitted during the study period, those with a diagnosis of DVT and taking anticoagulant therapy, those who refused to participate, and those with a length of stay of less than 24 h were excluded from the study. A consecutive sampling technique was used to select study participants.

### Operational definition

**Appropriate prophylaxis:** When pharmacological prophylaxis was administered to the patient when indicated and not contraindicated or when no pharmacological prophylaxis was given to a patient who was either not eligible or had an absolute contraindication.
**Inappropriate prophylaxis:** When pharmacological prophylaxis was not administered to the patient when indicated and not contraindicated or when pharmacological prophylaxis was given to a patient who was either not eligible or had an absolute contraindication.
**DVT prophylaxis indicated:** When the Padua risk score was ≥4 and the IMPROVE bleeding risk score was <7.
**DVT prophylaxis not indicated:** When the Padua risk score was <4, regardless of the IMPROVE bleeding risk score.
**DVT prophylaxis contraindicated:** When the IMPROVE bleeding risk score was ≥7.

### Data collection tools, procedures, and quality control

Data extraction tools were developed following a review of the literature (13, 17–21), with modifications made based on the type and context of patient medical data. These tools included participant sociodemographic details, pertinent laboratory findings, coagulation profile, length of hospital stay, diagnosis, number of diseases, the Padua assessment tool, and the IMPROVE bleeding risk score criteria. All of the information was obtained from the patients’ medical records. The format for data abstraction was pretested on 5% of the sample population, and any necessary modifications were made before the actual data collection period. Two pharmacists, who had received training on the study’s goals and fundamental data-gathering techniques, collected the data. Every day, the primary investigator checked the consistency, accuracy, and completeness of the data that had been gathered.

### Outcome measurements

Each patient’s risk of thromboembolism was assessed using the modified Padua risk assessment model. Each criterion was assigned a risk value between 1 and 4 based on the extent of its impact on the development of thromboembolism. The patient’s total risk score was determined by summing the points assigned to each Padua risk assessment parameter. Using the IMPROVE bleeding risk score criteria, the contraindications of DVT prophylaxis were evaluated. A total score of 7 or more was considered to indicate an absolute contraindication to prophylaxis. When a patient is hospitalized in a medical ward, stays for more than 24 h, and has no contraindications, a total score of 4 or more indicates a high risk of thromboembolism and qualifies them for pharmacologic prophylaxis (21, 22).

### Data processing and analysis

After the data collection, the data were entered into EpiData version 4.6, cleansed, and analyzed using STATA version 17. The results of the descriptive statistics were summarized using tables and figures. A Q–Q plot and a histogram were used to assess the normal distribution of the data. Depending on the distribution of the data, continuous variables were presented using the mean (standard deviation) and median (interquartile range), while categorical variables were presented using frequency and percent. After performing a Hosmer–Lemeshow goodness-of-fit test, a logistic regression model was used. A binary logistic regression analysis was conducted to identify the independent factors associated with the inappropriateness of DVT prophylaxis. Independent variables from the bivariate logistic regression analysis with a *p*-value of less than 0.2 were included in the multivariable logistic regression analysis to account for potential confounding. A *p*-value less than 0.05 was considered statistically significant.

### Research approval and the participants’ consent

The study was approved by the Debre Tabor University Institutional Research Ethics Review Committee (approval number DTU/Re/305/2016). The hospital’s medical director provided a letter of authorization, which was received by the medical ward director. Written informed consent was obtained from each respondent after they were informed of the study’s goals and purpose. Participants were informed that their participation in the study was voluntary and that their information would remain private and confidential. The names and addresses of the participants were excluded from the data abstraction format to protect their confidentiality. The study adhered to the Helsinki Declaration, ensuring that it was conducted in an anonymous and confidential manner.

## Results

### Sociodemographic characteristics of the participants

A total of 365 individuals participated in the study during the study period. The majority of the patients (54.5%) were male, and their mean (±SD) age was 46.4 ± 18.1 years. Payment for medical expenses was utilized by the great majority of the study participants (75.3%). Approximately one-fifth of the study participants were overweight (Table 1).

**Table 1:** Socio-demographic characteristics of the study participants (*N* = 365).

Variables	Categories	Frequency	Percentage	Mean ± SD
Sex	Male	199	54.5	
	Female	166	45.5	
Age	<41	171	46.8	46.4 ± 18.1
	41–64	116	31.8	
	≥65	78	21.4	
Marital status	Single	58	15.9	
	Married	284	77.8	
	Divorced	9	2.5	
	Widowed	14	3.8	
Religion	Orthodox	339	92.9	
	Muslim	21	5.7	
	Protestant	5	1.4	
Educational status	Unable to read and write	111	30.4	
	Primary	90	24.7	
	Secondary	104	28.5	
	College and above	60	16.4	
Occupational status	Daily labor	19	5.2	
	Farmer	208	57.0	
	Merchant	61	16.7	
	Government employee	24	6.6	
	Retired	53	14.5	
Body mass index (kg/m^2^)	<25	290	79.5	21.8 ± 3.3
	≥25	75	20.5	
Current drinker	Yes	246	67.4	
	No	119	32.6	
Place of residence	Urban	142	38.9	
	Rural	223	61.1	
Source of medicine	Free	90	24.7	
	Payment	275	75.3	
Monthly income	<3,000	182	49.9	3,200 (2300–7,000)
	>3,000	183	50.1	

### Clinical and laboratory profiles of the participants

According to the study, patients were most frequently diagnosed with infectious diseases (71.8%), followed by cardiovascular (57.3%) and gastrointestinal disorders (29.9%). The average length of hospital stay was 10.5 ± 4.5 days. In addition, 17.3% of patients were admitted to the intensive care unit (Table 2).

**Table 2:** Clinical and laboratory profile among study participants.

Variables	Categories	Frequency	Percentage	Mean ± SD
Diagnosis	Infectious diseases	262	71.8	
	Cardiovascular disease	209	57.3	
	Gastrointestinal disease	109	29.9	
	Renal disease	73	20.0	
	Endocrine/metabolic disease	56	15.3	
	Hematological disease	52	14.2	
	Rheumatoid disease	36	9.9	
	Respiratory disease	12	3.3	
	Neurological disease	9	2.5	
Number of diseases (comorbidity)	One	153		2 ± 2.6
	Two	107		
	Three	72		
	Four and above	51		
Length of hospitalization (days)	<7	86	23.6	10.5 ± 4.5
	≥7	279	76.4	
Admission room	ICU	63	17.3	
	Non-ICU	302	82.7	
Renal function test	Creatinine	365	100	1.2 ± 0.8
	BUN	347	95.1	21.4 ± 19.2
Liver function test	SGOT/AST	199	54.5	80.2 ± 39.4
	SGPT/ALT	195	53.4	61.5 ± 31.5
Coagulation profile	INR	55	15.1	1.5 ± 0.3
	Prothrombin time	53	14.5	15.41 ± 2.2
	aPTT	53	14.5	29.4 ± 5.9
Complete blood count	White blood cell	365	100	7.8 ± 3.7
	Neutrophil	365	100	6.3 ± 6.1
	Platelets	365	100	245.2 ± 120.1
Inflammatory marker	ESR	196	53.7	15.7 ± 13.7

NB: ICU, Intensive care unit; INR, international normalized ratio; aPTT, activated partial thromboplastin time; ESR, erythrocyte sedimentation rate; BUN, blood urea nitrogen; SGOT, serum glutamic oxaloacetic transaminase; SGPT, serum glutamic pyruvic transaminase.

### Risk factors for thromboembolisms

The common risk factors for thromboembolism included restricted mobility (40.0%), heart or respiratory failure (29.3%), acute infection and rheumatologic illness (86.5%), and other conditions (Table 3).

**Table 3:** Risk factors for thromboembolism based on the Padua assessment tool in the hospitalized patients.

DVT risk factor	Points given	Frequency	Percentages
Acute infection and rheumatologic disorder	1	315	86.3
Heart and/or respiratory failure	1	107	29.3
Acute myocardial infarction or ischemic stroke	1	102	27.9
Ongoing hormonal treatment	1	35	9.6
Elderly age ≥ 70	1	42	11.5
Recent (< 1 month) trauma and/or surgery	2	3	0.8
Reduced mobility	3	146	40.0
Active cancer	3	2	0.5
Already known thrombophilia condition	3	3	0.8
Previous VTE	3	8	2.2

VTE, venous thromboembolism.

### DVT risk stratification

The Padua assessment tool for thromboembolism was used in this study to determine the risk of DVT and design appropriate thromboprophylaxis. As a result, the classification of DVT risk was based on the sum of each specific risk factor. Consequently, 59.2% of the patients were classified as low risk. In terms of overall risk, the mean Padua score for the participants was 3.4 ± 2.1 (Table 4).

**Table 4:** DVT risk stratifications among the study participants.

Total risk factors	Risk stratification	Frequency (*n*)	Percentage (%)	Mean ± SD
0–3	Low	216	59.2	3.4 ± 2.1
≥4	High	149	40.8	

### Contraindications to thromboprophylaxis

There is a contraindication: patients with a total risk score of 4 or higher should receive thromboprophylaxis. The highest risk of bleeding was observed among individuals aged 65 years or older (21.4%) and those of the male sex (54.5%). A mean bleeding score of 8.5 ± 3.5 was obtained (Table 5).

**Table 5:** Contraindications for thromboprophylaxis according to the IMPROVE bleeding risk score criteria.

Factors	Categories	Points given	Frequency	Percentage	Mean ± SD
Age	≥65 years	3.5	78	21.4	46.4 ± 18.1
	40–64 years	1.5	116	31.8	
	<40 years	0	171	46.8	
Sex	Male	1	199	54.5	
	Female	0	166	45.5	
Renal function (ml/min/m^2^)	GFR ≥60	0	185	50.7	77.0 ± 56.0
	GFR 30–59	1	136	37.3	
	GFR <30	2.5	44	12.0	
Liver function	INR ≤ 1.5	0	33	9.1	
	INR > 1.5	2.5	22	6.0	
Platelets count	≥50 × 109/L	0	354	97.0	
	<50 × 109/L	4	11	3.0	
Admission to intensive care unit	Yes	2.5	63	17.3	
	No	0	302	82.7	
Central venous catheter	Yes	2	7	1.9	
	No	0	358	98.1	
Active gastric or duodenal ulcer	Yes	4.5	24	6.6	
	No	0	341	93.4	
Prior bleeding in the previous 3 months	Yes	4	8	2.2	
	No	0	357	97.8	
Rheumatic disease	Yes	2	36	9.9	
	No	0	329	90.1	
Active malignancy	Yes	2	6	1.6	
	No	0	359	98.4	
Bleeding risk score (the sum of all factors)	<7 (Low risk)		179	49.0	8.5 ± 3.5
	≥7 (High risk)		186	51.0	

### Appropriateness of thromboprophylaxis

**Figure 1:**
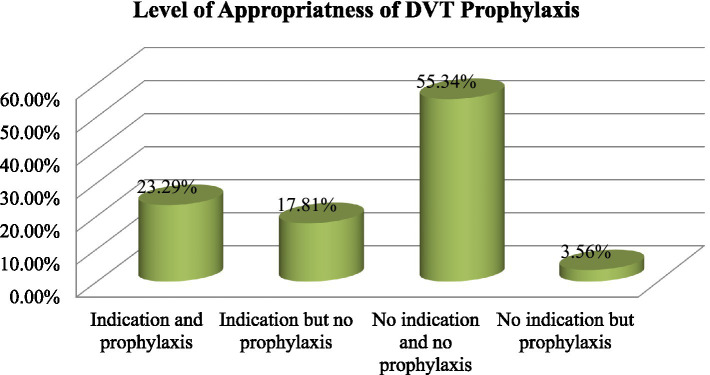
Appropriateness of DVT prophylaxis use among patients admitted to the medical ward.

Among the patients in our study, 21.37% received inappropriate thromboprophylaxis. Approximately 55.34% of the patients were those who were not eligible for thromboprophylaxis and who did not receive prophylaxis, while 23.29% were those who were eligible and received prophylaxis. The total of these two factors indicated the appropriateness of thromboprophylaxis (Figure 1).

### Prescribed anticoagulants for DVT prophylaxis

**Figure 2:**
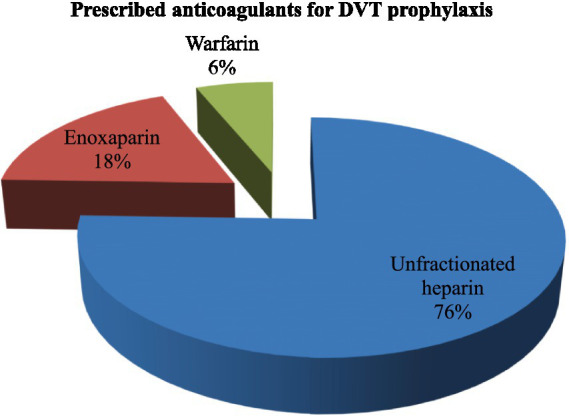
Prescribed anticoagulants for DVT prophylaxis (98).

The most frequently prescribed anticoagulants were unfractionated heparin (76%), a low molecular weight anticoagulant (enoxaparin) (18%), and warfarin (6%) (Figure 2).

### Interventions for inappropriate thromboprophylaxis

**Figure 3:**
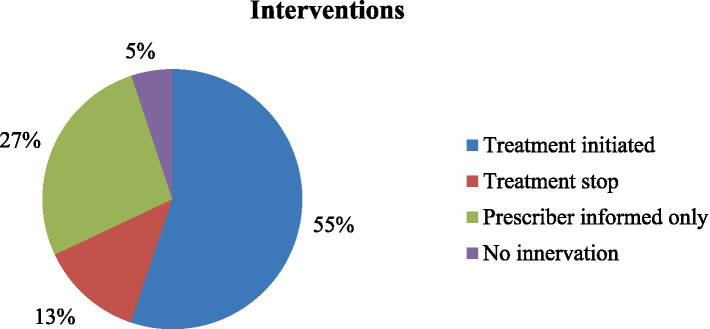
Interventions for the inappropriate use of DVT among patients admitted to the medical ward.

Following the discovery of the inappropriate use of thromboprophylaxis on the medical ward, interventions were implemented. Of the interventions provided, 55% were initiated for treatment, and 27% involved informing the prescribers only (Figure 3).

### Factors associated with inappropriate thromboprophylaxis use among the participants

According to the multivariate logistic regression analysis, the patients’ place of admission, length of stay, and type of doctor were significantly associated with the occurrence of inappropriate thromboprophylaxis use.

Thus, assuming all other variables remained constant, compared to the NICU patients, the patients admitted to the ICU were more likely to receive inappropriate thromboprophylaxis [AOR = 4.276, 95% CI: 1.878–16.134; *p* = ≤0.001]. Compared to the patients who stayed for less than 7 days, the patients who stayed for more than 6 days had a higher likelihood of receiving inappropriate thromboprophylaxis [AOR = 6.192, 95% CI: 2.085–14.391; *p* = ≤0.001]. In addition, it was observed that the patients receiving treatment from GPs were more likely to receive inappropriate thromboprophylaxis than those receiving care from specialists [AOR = 1.816, 95% CI: 1.007–3.207; *p* = 0.048] (Table 6).

**Table 6:** Factors associated with inappropriate thromboprophylaxis use among the participants.

Variables	Category	Thromboprophylaxis		COR	*p*-value	AOR	*p*-value
		Inappropriate	Appropriate				
Sex	Female	29	137	1		1	
	Male	49	150	1.543 (0.923–2.581)	0.098	1.187 (0.635–2.218)	0.591
Age	<41	33	138	1		1	
	41–64	24	92	1.091 (0.606–1.964)	0.772	0. 832 (0.409–1.692)	0.612
	≥65	21	57	1.541 (0.822–2.887)	0.177	1.155 (0.503–2.654)	0.734
BMI (kg/m^2^)	<25	55	235	1		1	
	≥25	23	52	1.889 (1.067–3.348)	0.029	1.953 (0.965–3.952)	0.063
GFR (ml/min/m^2^)	≥60	36	149	1		1	
	30–59	25	111	0.932 (0.529–1.642)	0.808	0.455 (0.179–1.155)	0.097
	<30	17	27	2.606 (1.284–5.288)	0.008	0.457 (0.194–1.078)	0.074
LoH (days)	<7	13	73	1		1	
	≥7	65	214	1.706 (0.888–3.274)	0.109	6.192 (2.085–14.391)	**≤0.001**
Place of admission	NICU	49	253	1		1	
	ICU	29	34	4.404 (2.460–7.883)	0.000	4.276 (1.878–16.134)	**≤0.001**
Place of residence	Urban	21	121	1		1	
	Rural	57	166	1.978 (1.139–3.437)	0.015	1.687 (0.866–3.283)	0.124
Thrombocytopenia	Yes	12	69	1		1	
	No	66	218	0.574 (0.293–1.125)	0.106	0.631 (0.292–1.361)	0.240
Drinker	Yes	61	185	1.978 (1.097–3.567)	0.023	1.657 (0.844–3.257)	0.143
	No	17	102	1		1	
Source of medicine	Free	15	75	1		1	
	Payment	63	212	1.486 (0.798–2.766)	0.212	1.534 (0.747–3.151)	0.244
Blood transfusion	Yes	67	14	0.253 (0.033 1.956)	0.188	0.287 (0.027–3.089)	0.304
	No	11	273	1		1	
Type of doctor	GP	26	149	1.852 (1.096–3.130)	0.021	1.816 (1.007–3.207)	**0.048**
	Specialist	52	138	1		1	

NB: BMI, body mass index; GFR, glomerular filtration rate; LOH, length of hospitalization; ICU, intensive care unit; COR, crude odds ratio; AOR, adjusted odds ratio; CI, confidence interval; *p*-value ≤0.05, ∗ *p*-value. The bold values in statistical significance.

## Discussion

Prophylactic treatment of deep vein thrombosis can save lives and prevent non-fatal symptomatic thromboembolism. It can also help avoid post-thrombotic syndrome, which is estimated to affect 15–40% of those who have had DVT in the past (23). These outcomes are highly beneficial in terms of human resources. Therefore, patients in our hospitals should receive careful evaluation regarding the prophylaxis of deep vein thrombosis. Many patients who are at significant risk of complications do not receive thromboprophylaxis, despite its potential benefits. If thromboprophylaxis is not contraindicated, patients with a total risk score higher than 4 should be studied. In our findings, the most common absolute contraindications that excluded the patients from receiving thromboprophylaxis included being older than 65 years, ICU admission, and a GFR of less than 30 mL/min/a. Nonetheless, a study conducted by the American University of Beirut Medical Center found that the most common contraindication was renal impairment (24).

The current study found that 17.81% of eligible patients did not receive thromboprophylaxis, which is consistent with an Iranian study reporting 18.3% (7). However, compared to a study conducted in Australia, only 23% of patients in the medium-risk group and 5% in the high-risk group received the recommended preventive treatment (23). The inability to accurately classify patients into the appropriate risk group and the difficulty in selecting appropriate prophylaxis for a specific risk group are key factors contributing to inappropriate thromboprophylaxis. Another reason thromboprophylaxis is not administered is that patients with a high risk of DVT may have multiple diagnoses, with the primary and major diagnoses taking priority over the risk of DVT in each individual patient. Clinicians’ lack of awareness of DVT prophylactic protocols and DVT risk stratification may also contribute to the underuse of thromboprophylaxis (23, 25).

According to the results of the current investigation, thromboprophylaxis regimens were administered improperly to approximately 21.37% of patients after 365 patients were assessed using the Padua assessment tool. Similar low rates were also noted in research conducted in other Asian countries (26, 27) and Iran (7). However, the rate in our study was lower than those of previous studies conducted in California (28), Brazilian Society (29), and sub-Saharan Africa (30). Some studies have also indicated that venous thromboembolism remains the primary cause of unexpected mortality, despite patients having received appropriate prophylaxis (19). This discrepancy might result from the fact that our study exclusively focused on pharmacologic prophylaxis, while the prior study evaluated the use of both pharmaceutical and mechanical prophylaxis. Furthermore, although our study used the Padua assessment tool, other studies also used alternative assessment techniques.

Our investigation revealed that the place of the patient’s admission, the duration of their hospital stay, and the types of physicians they saw were the main contributors to the improper use of thromboprophylaxis. As a result, individuals who spent 7 days or more in the hospital had a 6-fold increased risk of inappropriate thromboprophylaxis use compared to those who stayed for fewer days. These findings are in line with those of previous studies (31, 32). As a result, sitting still may make it difficult to move the legs and may even cause compression, reducing blood flow to the legs. Remarkably, the group classified as having sedentary professions had a higher risk of DVT, which medical professionals may overlook or fail to notice if patients remain in the hospital for an extended period. Due to bed shortages, extended stays in acute hospitals hinder patient flow and access to care while also increasing the risk of hospital-acquired illnesses. The shortage of hospital beds raises concerns about patient safety and the adequacy of the healthcare system’s infrastructure (33).

The hospital’s place of admission was another factor contributing to the incidence of improper thromboprophylaxis usage. Consequently, patients admitted to the ICU had approximately a 4-fold higher risk of inappropriate DVT prophylaxis use compared to those admitted to non-ICUs. Inappropriate use of DVT prophylaxis in the ICU has also been reported in previous studies (34–36). Since DVT is often clinically silent in the ICU, especially in patients who are sedated and on mechanical ventilation, the majority of patients admitted there do not receive the recommended thromboprophylaxis. As ICU-acquired thromboembolic events may resemble a variety of other illnesses, they are challenging to detect (37).

In our study, the individuals treated by specialists received appropriate DVT prophylaxis at a rate approximately double that of those treated by general practitioners. This result is consistent with research showing that specialists, compared to general practitioners, typically exhibit better adherence to guidelines, ensuring the proper use of prophylactics (38). One possible explanation for GPs’ improper use of thromboprophylaxis is a lack of awareness and familiarity with evidence-based practices. A problem frequently noted in the sub-Saharan African context is the severe shortage of experts in healthcare systems in low-resource environments, such as Ethiopia, which forces general practitioners to handle complex situations without the necessary resources or experience (39). General practitioners may be encouraged to use prophylaxis in low- to high-risk patients, for whom the risks may outweigh the benefits, using performance indicators that promote DVT prophylaxis for all medical patients. However, specialists are better able to identify people who will actually benefit from it (40). Since similar approaches have significantly enhanced DVT prophylaxis in low-resource healthcare systems, it is recommended that GPs participate in ongoing professional development programs and systematically incorporate thromboprophylaxis guidelines into their daily practices to close these gaps (41).

Our investigation revealed that the supply of interventions resolved 95% of the problems associated with inappropriate deep vein thromboprophylaxis use, a finding supported by previous studies (7, 42), which demonstrated that clinical pharmacy interventions are statistically significant in preventing the improper use of DVT prophylaxis. Another study conducted in Belgium (43) reported that pharmacist-driven interventions increased the percentage of critically ill medical patients receiving VTE prophylaxis, with benefits that persisted over time. Compared to education-based approaches, there was a notable improvement during the pharmacist-intervention phase. It is more effective to use clinical pharmacy interventions, particularly when it comes to maintaining optimum thromboprophylaxis (44, 45). Clinical pharmacists can help healthcare professionals apply antithrombotic prophylaxis and medication use rationally in hospitals by using various risk assessment tools and providing support.

### Strengths and limitations

One potential strength of this study is its observational follow-up study design. However, when interpreting the study findings, the following limitations should be considered. The results of this study are limited to one location and cannot be generalized to all hospitals in Ethiopia. In addition, it omitted information regarding the appropriateness of prophylaxis in terms of dosage and treatment duration. Furthermore, based on the sample size estimate, we did not include enough patients to achieve the desired statistical power.

## Conclusion

In our study, the appropriateness of DVT prophylaxis use was suboptimal, especially among the patients treated by general practitioners, those hospitalized in the intensive care unit, and those confined to the ward for longer than a few days. Using an integrated risk stratification checklist is an effective way to globally increase the use of DVT prophylaxis. Staff members working in medical wards should follow protocols and possess an understanding of thromboprophylaxis and DVT risk factors.

## Data Availability

The original contributions presented in the study are included in the article/supplementary material, further inquiries can be directed to the corresponding author.

## References

[1] Stone J Hangge P Albadawi H Wallace A Shamoun F Knuttien MG. Deep vein thrombosis: pathogenesis, diagnosis, and medical management. Cardiovasc Diagn Ther. (2017) 7:S276–84. doi: 10.21037/cdt.2017.09.01, PMID: 29399531 PMC5778510

[2] Geerts WH Bergqvist D Pineo GF Heit JA Samama CM Lassen MR. Prevention of venous thromboembolism: American College of Chest Physicians evidence-based clinical practice guidelines. Chest. (2008) 133:381S–453S. doi: 10.1378/chest.08-0656, PMID: 18574271

[3] Van Wicklin SA Ward KS Cantrell SW. Implementing a research utilization plan for prevention of deep vein thrombosis. AORN J. (2006) 83:1351–62. doi: 10.1016/S0001-2092(06)60149-X, PMID: 16821673

[4] Giri S Singh A Varghese J Ingawale S Roy A. Outcome of pharmacological thromboprophylaxis in hospitalized patients with cirrhosis–a systematic review and meta-analysis. Eur J Gastroenterol Hepatol. (2023) 35:674–81. doi: 10.1097/MEG.0000000000002564, PMID: 37115994

[5] Onwuzo C Olukorode J Sange W Tanna SJ Osaghae OW Hassan A. A review of the preventive strategies for venous thromboembolism in hospitalized patients. Cureus. (2023) 15:e48421. doi: 10.7759/cureus.48421, PMID: 38074047 PMC10701607

[6] Samama MM Cohen AT Darmon JY Desjardins L Eldor A Janbon C. A comparison of enoxaparin with placebo for the prevention of venous thromboembolism in acutely ill medical patients. N Engl J Med. (1999) 341:793–800. doi: 10.1056/NEJM199909093411103, PMID: 10477777

[7] Khalili H Dashti-Khavidaki S Talasaz AH Mahmoudi L Eslami K Tabeefar H. Is deep vein thrombosis prophylaxis appropriate in the medical wards? A clinical pharmacists' intervention study. Pharm World Sci. (2010) 32:594–600. doi: 10.1007/s11096-010-9412-y, PMID: 20623385

[8] Kahn SR Lim W Dunn AS Cushman M Dentali F Akl EA. Prevention of VTE in nonsurgical patients: antithrombotic therapy and prevention of thrombosis: American College of Chest Physicians Evidence-Based Clinical Practice Guidelines. Chest. (2012) 141:e195S–226S. doi: 10.1378/chest.11-2296, PMID: 22315261 PMC3278052

[9] Guyatt GH Akl EA Crowther M Gutterman DD Schuünemann HJ. Executive summary: antithrombotic therapy and prevention of thrombosis: American College of Chest Physicians evidence-based clinical practice guidelines. Chest. (2012) 141:7S–47S. doi: 10.1378/chest.1412S3, PMID: 22315257 PMC3278060

[10] Kahn SR Panju A Geerts W Pineo GF Desjardins L Turpie AG. Multicenter evaluation of the use of venous thromboembolism prophylaxis in acutely ill medical patients in Canada. Thromb Res. (2007) 119:145–55. doi: 10.1016/j.thromres.2006.01.011, PMID: 16516275

[11] Cohen AT Tapson VF Bergmann JF Goldhaber SZ Kakkar AK Deslandes B. Venous thromboembolism risk and prophylaxis in the acute hospital care setting (ENDORSE study): a multinational cross-sectional study. Lancet. (2008) 371:387–94. doi: 10.1016/S0140-6736(08)60202-0, PMID: 18242412

[12] Greene MT Spyropoulos AC Chopra V Grant PJ Kaatz S Bernstein SJ. Validation of risk assessment models of venous thromboembolism in hospitalized medical patients. Am J Med. (2016) 129:1001.e9. e18. doi: 10.1016/j.amjmed.2016.03.031 27107925

[13] Badireddy M. Mudipalli V.R., Deep venous thrombosis prophylaxis. In: StatPearls. Treasure Island (FL): StatPearls Publishing. (2023). 30521286

[14] Ghanima W Wik HS Tavoly M Enden T Jelsness-Jørgensen LP. Late consequences of venous thromboembolism: measuring quality of life after deep vein thrombosis and pulmonary embolism. Thromb Res. (2018) 164:170–6. doi: 10.1016/j.thromres.2017.07.025, PMID: 28760416

[15] Dominguez-Vicent A Brautaset R Venkataraman AP. Repeatability of quantitative measurements of retinal layers with SD-OCT and agreement between vertical and horizontal scan protocols in healthy eyes. PLoS One. (2019) 14:e0221466. doi: 10.1371/journal.pone.0221466, PMID: 31437222 PMC6705867

[16] Masters R Powers D. Clarifying assumptions in age-period-cohort analyses and validating results. PLoS One. (2020) 15:e0238871. doi: 10.1371/journal.pone.0238871, PMID: 33021978 PMC7537862

[17] Ayalew MB Horsa BA Zeleke MT. Appropriateness of pharmacologic prophylaxis against deep vein thrombosis in medical wards of an Ethiopian referral hospital. J Vasc Med. (2018) 2018:1–7. doi: 10.1155/2018/8176898, PMID: 30105097 PMC6076918

[18] Mohammed AS Taha NM Abdel-Aziz EM. Nurses' performance regarding venous thromboembolism prophylaxis at intensive care unit. Zagazig Nurs J. (2018) 14:1–17. doi: 10.21608/znj.2018.37454

[19] Shah SS Abdi A Özcem B Basgut B. The rational use of thromboprophylaxis therapy in hospitalized patients and the perspectives of health care providers in northern Cyprus. PLoS One. (2020) 15:e0235495. doi: 10.1371/journal.pone.0235495, PMID: 32667938 PMC7363080

[20] Yap DFS Ng ZY Wong CY Saifuzzaman MM Yang L. Appropriateness of deep vein thrombosis (DVT) prophylaxis use among medical inpatients: a DVT risk alert tool (DRAT) study. Med J Malaysia. (2019) 74:45. 30846662

[21] Rosenberg DJ Press A Fishbein J Lesser M McCullagh L McGinn T. External validation of the IMPROVE bleeding risk assessment model in medical patients. Thromb Haemost. (2016) 116:530–6. doi: 10.1160/TH16-01-0003 27307054

[22] Lai J. Venous thromboembolism prophylaxis adult inpatient/ambulatory Clinical Practice Guideline (2014). Available at: https://acforum-excellence.org/Resource-Center/resource_files/1585-2020-07-01-062736.pdf

[23] Ahmad HA Geissler A MacLellan DG. Deep venous thrombosis prophylaxis: are guidelines being followed? ANZ J Surg. (2002) 72:331–4. doi: 10.1046/j.1445-2197.2002.02402.x 12028089

[24] Masroujeh R Shamseddeen W Isma’eel H Otrock ZK Khalil IM Taher A. Underutilization of venous thromboemoblism prophylaxis in medical patients in a tertiary care center. J Thromb Thrombolysis. (2008) 26:138–41. doi: 10.1007/s11239-007-0084-y, PMID: 17701104

[25] Rahim SA Panju A Pai M Ginsberg J. Venous thromboembolism prophylaxis in medical inpatients: a retrospective chart review. Thromb Res. (2003) 111:215–9. doi: 10.1016/j.thromres.2003.09.010, PMID: 14693166

[26] Pinjala R. Venous thromboembolism risk & prophylaxis in the acute hospital care setting (ENDORSE), a multinational cross-sectional study: results from the Indian subset data. Indian J Med Res. (2012) 136:60–7. PMID: 22885265 PMC3461719

[27] Nekoonam B Eshraghi A Hajiesmaeili M Sahraei Z. Deep vein thrombosis prophylaxis evaluation in intensive care unit. Arch Crit Care Med. (2016) 1:e8497. doi: 10.17795/accm-8497

[28] White RH. The epidemiology of venous thromboembolism. Circulation. (2003) 107:I-4–8. doi: 10.1161/01.CIR.0000078468.11849.66, PMID: 12814979

[29] Pereira CA Brito SS Martins AS Almeida CM. Deep venous thrombosis prophylaxis: practical application and theoretical knowledge in a general hospital. J Vasc Bras. (2008) 7:18–27. doi: 10.1590/S1677-54492008000100005

[30] Kingue S Bakilo L Ze Minkande J Fifen I Gureja YP Razafimahandry HJC. Epidemiological African day for evaluation of patients at risk of venous thrombosis in acute hospital care settings: cardiovascular topic. Cardiovasc J Afr. (2014) 25:159–64. doi: 10.5830/CVJA-2014-025, PMID: 25192298 PMC4170174

[31] Mahan CE Fisher MD Mills RM Fields LE Stephenson JJ Fu AC. Thromboprophylaxis patterns, risk factors, and outcomes of care in the medically ill patient population. Thromb Res. (2013) 132:520–6. doi: 10.1016/j.thromres.2013.08.013, PMID: 24080150

[32] de Bastos M Barreto SM Caiafa JS Bogutchi T Rezende SM. Assessment of characteristics associated with pharmacologic thromboprophylaxis use in hospitalized patients: a cohort study of 10 016 patients. Blood Coagul Fibrinolysis. (2013) 24:691–7. doi: 10.1097/MBC.0b013e328360a52c, PMID: 24047889

[33] Maguire PA Taylor IC Stout RW. Elderly patients in acute medical wards: factors predicting length of stay in hospital. Br Med J (Clin Res Ed). (1986) 292:1251–3. doi: 10.1136/bmj.292.6530.1251, PMID: 3085801 PMC1340254

[34] Permpikul C Chaiyasoot W Panitchote A. Incidence of proximal deep vein thrombosis in medical critical care patients. Thromb J. (2022) 20:5. doi: 10.1186/s12959-022-00363-5, PMID: 35123485 PMC8817527

[35] Malato A Dentali F Siragusa S Fabbiano F Kagoma Y Boddi M. The impact of deep vein thrombosis in critically ill patients: a meta-analysis of major clinical outcomes. Blood Transfus. (2015) 13:559–68. doi: 10.2450/2015.0277-14 26513770 PMC4624530

[36] Khouli H Shapiro J Pham VP Arfaei A Esan O Jean R. Efficacy of deep venous thrombosis prophylaxis in the medical intensive care unit. J Intensive Care Med. (2006) 21:352–8. doi: 10.1177/0885066606292880 17095499

[37] Minet C Potton L Bonadona A Hamidfar-Roy R Somohano CA Lugosi M. Venous thromboembolism in the ICU: main characteristics, diagnosis and thromboprophylaxis. Crit Care. (2015) 19:1–9. doi: 10.1186/s13054-015-1003-9 26283414 PMC4539929

[38] Vallano A Arnau JM Miralda GP Pérez-Bartolí J. Use of venous thromboprophylaxis and adherence to guideline recommendations: a cross-sectional study. Thromb J. (2004) 2:3. doi: 10.1186/1477-9560-2-3, PMID: 15059286 PMC416491

[39] Naicker S Eastwood JB Plange-Rhule J Tutt RC. Shortage of healthcare workers in sub-Saharan Africa: a nephrological perspective. Clin Nephrol. (2010) 74:S129–33. doi: 10.5414/CNP74S129, PMID: 20979978

[40] Berhe DF Taxis K Haaijer-Ruskamp FM Mol PGM. Healthcare professionals' level of medication knowledge in Africa: a systematic review. Br J Clin Pharmacol. (2018) 84:2729–46. doi: 10.1111/bcp.13746, PMID: 30171617 PMC6256006

[41] Mokadem NE El-Sayed S, Effect of educational intervention on critical care Nurses' adherence to the clinical practice guidelines for preventing venous thromboembolism in critically ill patients. American Journal of Nursing. (2019). 7:974–982. doi: 10.12691/ajnr-7-6-10

[42] Cohn SL Adekile A Mahabir V. Improved use of thromboprophylaxis for deep vein thrombosis following an educational intervention. J Hosp Med. (2006) 1:331–8. doi: 10.1002/jhm.137, PMID: 17219525

[43] Vervacke A Lorent S Motte S. Improved venous thromboembolism prophylaxis by pharmacist-driven interventions in acutely ill medical patients in Belgium. Int J Clin Pharm. (2014) 36:1007–13. doi: 10.1007/s11096-014-9988-8, PMID: 25087039

[44] Kiracı ZK Yalçın N Cennet Ö Demirkan K Yorgancı K. Education and clinical pharmacist-led management strategies for the risk and prophylaxis of venous thromboembolism in general surgery. Thromb J. (2023) 21:86. doi: 10.1186/s12959-023-00530-2, PMID: 37559115 PMC10413499

[45] Mokhtari M Attarian H Norouzi M Kouchek M Kashani BS Sirati F. Venous thromboembolism risk assessment, prophylaxis practices and interventions for its improvement (AVAIL-ME extension project, Iran). Thromb Res. (2014) 133:567–73. doi: 10.1016/j.thromres.2014.01.006, PMID: 24507872

